# BmDJ-1 Is a Key Regulator of Oxidative Modification in the Development of the Silkworm, *Bombyx mori*

**DOI:** 10.1371/journal.pone.0017683

**Published:** 2011-03-24

**Authors:** Hiroko Tabunoki, Hiroaki Ode, Yutaka Banno, Susumu Katsuma, Toru Shimada, Kazuei Mita, Kimiko Yamamoto, Ryoichi Sato, Reiko Ishii-Nozawa, Jun-ichi Satoh

**Affiliations:** 1 Department of Bioinformatics and Molecular Neuropathology, Meiji Pharmaceutical University, Tokyo, Japan; 2 The Center of Genetic Resources, University of Kyushu, Fukuoka, Japan; 3 Department of Agricultural and Environmental Biology, Graduate School of Agricultural and Life Sciences, The University of Tokyo, Tokyo, Japan; 4 Insect Genome Laboratory, National Institute of Agrobiological Sciences, Tsukuba, Japan; 5 Bio-Applications and Systems Engineering, Tokyo University of Agriculture and Technology, Koganei, Tokyo, Japan; 6 Department of Clinical Pharmacology, Meiji Pharmaceutical University, Tokyo, Japan; Newcastle University, United Kingdom

## Abstract

We cloned cDNA for the *Bombyx mori* DJ-1 protein (BmDJ-1) from the brains of larvae. BmDJ-1 is composed of 190 amino acids and encoded by 672 nucleotides. Northern blot analysis showed that BmDJ-1 is transcribed as a 756-bp mRNA and has one isoform. Reverse transcriptase (RT)-PCR experiments revealed that the BmDJ-1 was present in the brain, fatbody, Malpighian tubule, ovary and testis but present in only low amounts in the silkgland and hemocyte of day 4 fifth instar larvae. Immunological analysis demonstrated the presence of BmDJ-1 in the brain, midgut, fatbody, Malpighian tubule, testis and ovary from the larvae to the adult. We found that BmDJ-1 has a unique expression pattern through the fifth instar larval to adult developmental stage. We assessed the anti-oxidative function of BmDJ-1 using rotenone (ROT) in day 3 fifth instar larvae. Administration of ROT to day 3 fifth instar larvae, together with exogenous (BmNPV-BmDJ-1 infection for 4 days in advance) BmDJ-1, produced significantly lower 24-h mortality in BmDJ-1 groups than in the control. 2D-PAGE revealed an isoelectric point (pI) shift to an acidic form for BmDJ-1 in BmN4 cells upon ROT stimulus. Among the factors examined for their effects on expression level of BmDJ-1 in the hemolymph, nitric oxide (NO) concentration was identified based on dramatic developmental stage-dependent changes. Administration of isosorbide dinitrate (ISDN), which is an NO donor, to BmN4 cells produced increased expression of BmDJ-1 compared to the control. These results suggest that BmDJ-1 might control oxidative stress in the cell due to NO and serves as a development modulation factor in *B. mori*.

## Introduction

The protein DJ-1 is ubiquitously expressed in cells and it is highly conserved across a wide variety of organisms, showing moderate sequence identity with heat shock protein 31 (HSP31) chaperones and ThiJ/PfpI cysteine proteases [1]. Mutated forms of DJ-1 are known to cause early onset autosomal recessive juvenile Parkinson's disease (PD), and many studies have demonstrated a neuro-protective role of DJ-1. DJ-1, which is encoded by PARK7, is a multi-functional protein that plays roles in chaperoning, RNA-binding, SUMOylation, apoptosis, and protease activity [2].

Additionally, DJ-1 is induced by oxidative modification and is rapidly oxidized at position Cys 106 [3]. Oxidative modification leads to mitochondrial damage in cultured cells exposed to 1-methyl-4-phenyl-1,2,3,6 tetrahydropyridine (MPTP), 6-hydroxydopamine (6-OHDA), paraquat (PQ), and rotenone (ROT), which inhibit the mitochondrial electron transfer chain of mitochondrial complex I [4]. These compounds enhance production of reactive oxygen species (ROS) and reduce production of ATP, resulting in mitochondria dysfunction [5]. DJ-1 seems to directly scavenge free radicals from mitochondria in response to these oxidative stresses. MPTP, 6-OHDA, PQ, and ROT are used to produce PD models in rats and *Drosophila* and to analyze the pathology of PD [6], [7].

DJ-1 has a dimer structure, and the L166P mutation produces structural perturbation that causes the protein to be ubiquitinated and susceptible to degradation by the 26S proteasome, significantly reducing its half-life *in vivo* [8], [9]. L166P DJ-1 forms unstable dimers with disrupted protein folding and function [10]. The C106A mutation results in a loss-of-function of DJ-1 protease and chaperone activity [11], [12]. However, the precise pathology due to mutations and species-specific biological functions of DJ-1 remain unclear.

The silkworm, *Bombyx mori*, a Lepidopteran insect, has been utilized as a model system for basic science research because of its well-characterized genome, availability of various genetic mutants, and the development of transgenic, RNAi, and microarray technologies [13], [14], [15], [16], [17]. The complete silkworm genome has approximately 18,510 genes, including a substantial number of mammalian orthologs [13], [16].

In the present study, we cloned the silkworm *B. mori* DJ-1 ortholog (BmDJ-1), clarified its expression pattern during development, and examined its anti-oxidative function. BmDJ-1 is a newly identified member of the DJ-1 family and is a growth-associated protein that is altered with development in *B. mori*.

## Results

### Molecular cloning of BmDJ-1

Amplifying BmDJ-1 by RT-PCR with 5′ RACE using gene-specific primers from *B. mori* larvae brain cDNA produced an 86-bp product. The Kozak consensus sequence AAAATGAAG [18] was found to be present at the site of translation initiation determined using NetStart software [19]. Therefore, we determined that the cDNA encoded a putative 5′-untranslated sequence of 95 bp, an ATG start site, and an open reading frame (ORF) at position 96 extending to position 668. The deduced ORF of BmDJ-1 was composed of 672 nucleotides comprising 190 amino acids, had a molecular weight of 20,113 Dalton, and a putative isoelectric point (pI) of 5.15.

The nucleotide sequence reported in this paper has been submitted to GeneBank/DDBJ SAKURA Data bank Accession No. AB281053.

A computer search of the SMART database (http://smart.embl-heidelberg.de/) revealed that BmDJ-1 contained a DJ-1_PfpI domain at position 31T-173T. We identified the location of the BmDJ-1 gene in scaffold 2995719-2998746 of chromosome 23 at the splitting of 5 blocks by linkage mapping 28 chromosomes by SNP markers [20].

**Figure 1:**
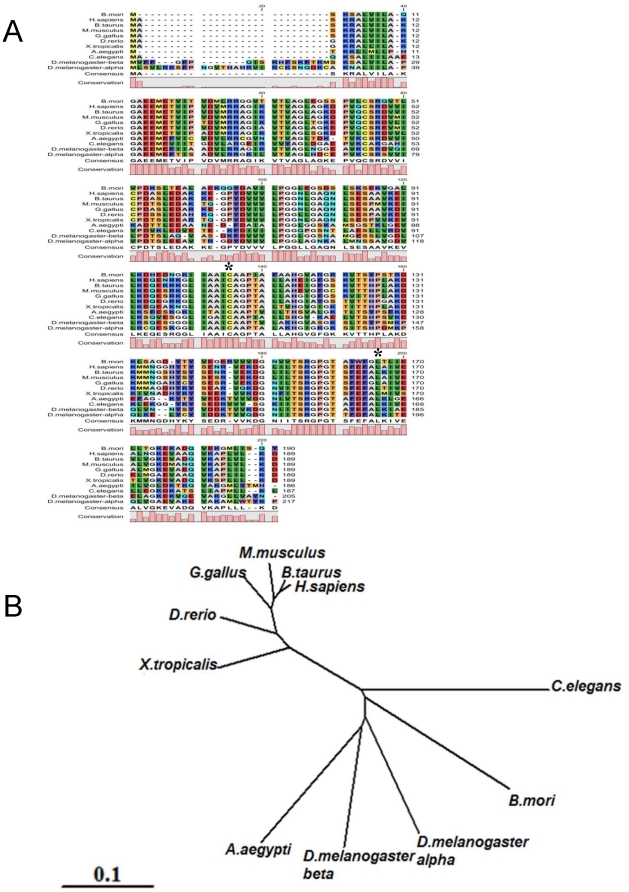
Alignment and Phylogenic tree of *B. mori* DJ-1 with other DJ-1 proteins. A. Conserved amino acid sequences C at position 106 and L at position 166 of human DJ-1 are marked by asterisks (*). The level of conserved amino acid residues among the various species are graphically shown below the sequences. The residues of the alignment are color-coded according to the Rasmol color scheme (http://life.nthu.edu.tw/~fmhsu/rasframe/COLORS.HTM#aminocolors) B. The unrooted bootstrap tree of *B. mori* DJ-1 and DJ-1 protein of other species. Sequences are *Homo sapiens*, *Bos Taurus*, *Mus musculus*, *Gallus gallus*, *Danio rerio*, *Xenopus tropicalis*, *Drosophila melanogaster*-α and -β, *Caenorhabditis elegans*, and *Bombyx mori* DJ-1.

A BLAST search showed that BmDJ-1 has 50% amino acid sequence identity to *D. rerio* DJ-1 (NCBI gene ID:449674) and *D. melanogaster* DJ-1 beta (NCBI gene ID:43652); 47% amino acid sequence identity to *H. sapiens* (NCBI gene ID:11315), *X. tropicalis* (NCBI gene ID:548568), *G. gallus* (NCBI gene ID:395227), *B. taurus* (NCBI gene ID:511268), and *R. novaltis* (NCBI gene ID:117287) DJ-1; 46% amino acid sequence identity to *C. elegans* (NCBI gene ID:183625) and *M. musculus* (NCBI gene ID:57320) DJ-1; and 45% amino acid sequence identity to *D. melanogaster* DJ-1 alpha (NCBI gene ID:36543). An alignment of the deduced BmDJ-1 amino acid sequences and DJ-1 orthologs from other species using CLC Work Bench 3.2.3 showed that the BmDJ-1 protein sequence contains all of the conserved Cys and Leu residues (Fig. 1-A, black asterisks).

The phylogenetic tree placed *D. melanogaster* DJ-1 and BmDJ-1 into a distinct cluster (Fig. 1-B).

### BmDJ-1 mRNA is expressed in various tissues in fifth instar larvae

**Figure 2:**
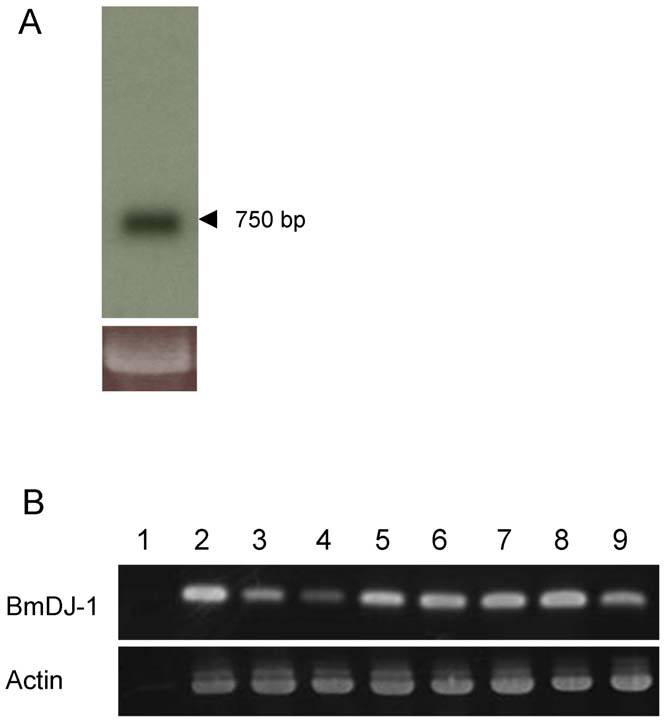
Northern blot analysis and RT-PCR of BmDJ-1. A. Total RNA isolated from *B. mori* ovary was analyzed by northern blot analysis using a BmDJ-1 probe. A band at about 756 bp was identified as the BmDJ-1 transcript. The amount of total RNA is 12 µg per lane. 18S ribosomal RNA was used as a control for monitoring RNA loading. B. RT-PCR for BmDJ-1 from cDNA samples synthesized from diverse larval tissues. RT-PCR for actin was used as a positive loading control and RT-PCR reaction, without reverse transcriptase, was used as a negative control. Lane 1, brain (RT-); lane 2, brain; lane 3, midgut; lane 4, silk gland; lane 5, fat body; lane 6, Malpighian tubule; lane 7, ovary; lane 8, testis; lane 9, hemocyte.

Northern blot analysis revealed that there is a single transcription product for BmDJ-1 with a size of 756 bp (Fig. 2A).

We used RT-PCR to investigate the expression profile of BmDJ-1 mRNA in various tissues. BmDJ-1 showed high expression in the brain, fatbody, Malpighian tubule, ovary, and testis (Fig. 2B, upper panel lanes 2, 5–8) and low expression in the midgut, silkgland, and hemocyte (Fig. 2B, upper panel lanes 3, 4, 9).

### Specificity of antibody against BmDJ-1

**Figure 3:**
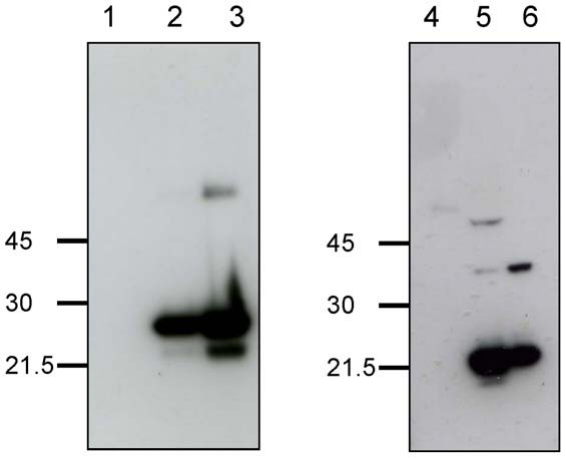
Specificity of anti-BmDJ-1 antibody. The recombinant protein, cell or tissue lysate were separated on a 12% SDS-PAGE gel, transferred onto a nitrocellulose membrane, and processed for immunoblotting with anti-BmDJ-1 antibody. The following samples were loaded in each lane: 1, 1 µg recombinant CBP as a negative control; 2, 0.25 µg recombinant BmDJ-1 protein; 3, 0.5 µg recombinant BmDJ-1 protein; 4, HEK 293 cells; 5, BmN4 cells; 6, larvae brain.

We examined the utility of anti-BmDJ-1 antibodies raised against the recombinant Xpress-tagged BmDJ-1 to identify BmDJ-1. Anti-BmDJ-1 antibody reacted with both recombinant BmDJ-1 protein as a 25-KDa band and BmDJ-1 in the cell and tissue lysate from *B. mori* as a 20-KDa band. In contrast, BmDJ-1 antibodies did not recognize recombinant carotenoid binding protein (CBP) from *B. mori* tagged with GST [21] and HEK 293 cell lysate (Fig. 3, lanes 1 and 4). The molecular weight of the recombinant BmDJ-1 protein (Fig. 3, lanes 2 and 3) was slightly greater than the endogenous BmDJ-1 protein (Fig. 3, lanes 5 and 6), excluding the possibility of non-specific binding to the Xpress tag.

### Identification of developmental stage and tissue-specific expression patterns of BmDJ-1 by immunoblotting

**Figure 4:**
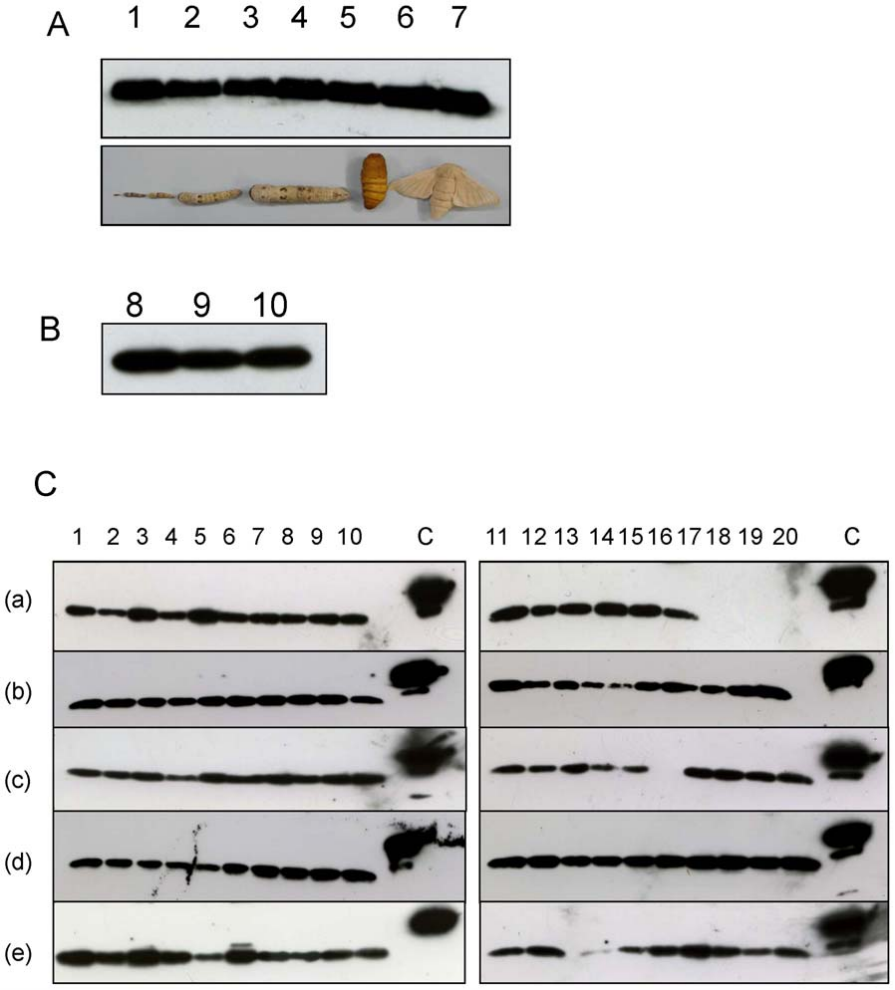
Developmental and tissue distribution of BmDJ-1 in *B. mori*. A. Aliquots (5 µg) of whole body homogenates from day 0 larvae of the first (lane 1), second (lane 2), third (lane 3), fourth (lane 4), and fifth (lane 5) instars, the pupae (lane 6), and the adult (lane 7) were separated by SDS-PAGE, transferred to nitrocellulose, and probed with anti-BmDJ-1 antibody. B, Aliquots (5 µg) of brain of the fifth instar larvae (lane 8), pupae (lane 9), and adults (lane 10). C. Aliquots (5 µg) of protein of various tissues were subjected to SDS-PAGE and examined for BmDJ-1 expression. The following tissues are shown: a, midgut; b, fatbody; c, Malpighian tubule; d, testis; and e, ovary were isolated from day 0 to 12 fifth instar larvae (lanes 1 to 13), from day 0, 1, 3, 4, 7 and 8 pupae (lanes 14 to 19), and from day 0 adults (lane 20). No samples were loaded in panel a, lanes 17, 18, 19, 20; panel c, lane 16; and panel e, lane 13.

Distribution of BmDJ-1 expression by developmental stage and tissue is shown in Figure 4. Whole body expression is roughly equal for all larval instars, pupae, and adults (Fig. 4A and S1A, lanes 1–7). Moreover, equal amounts of BmDJ-1 are found in the brains of fifth instar larvae, pupae, and adults, but it is slightly increased in larvae (Fig. 4B and S1B, lanes 8–10). To determine the distribution pattern of BmDJ-1, we studied tissues (midgut, fatbody, Malpighian tubule, ovary, and testis; Fig. 4C and S1C) from day 0 fifth instar larvae to adults by immunoblotting. BmDJ-1 was expressed in the larval through adult developmental stages in these tissues, but expression was low in day 1 pupae (fatbody, Malpighian tubule and ovary; Fig. 4C, panels b, c, e, lane 14). Expression levels increased with pupal stadium from day 0 to 4 (Fig. 4C, panels f–h) and high levels of BmDJ-1 expression were also identified in the testis during these developmental stages (Fig. 4C, panel q). Therefore, BmDJ-1 showed a unique day-to-day expression pattern from day 0 fifth instar larvae to the adult developmental stages.

### The pI of BmDJ-1 shifted acidic by ROT stimulation

**Figure 5:**
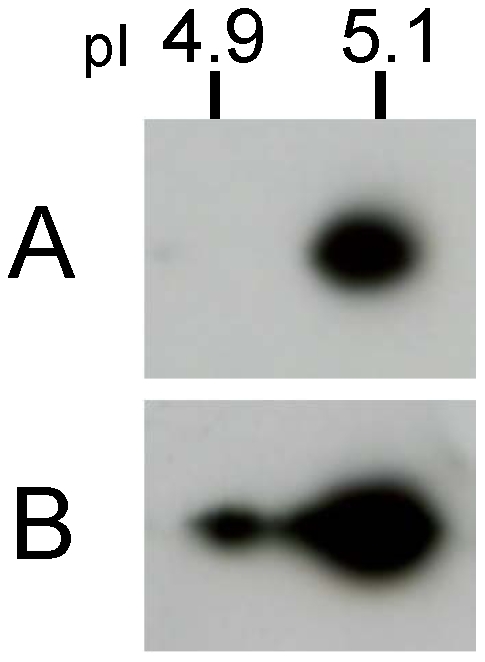
Effect of BmDJ-1 on ROT-induced oxidative stress in BmN4 cells. A. BmN4 cells exposed to ROT for 3 h were examined for BmDJ-1 content by 2D-PAGE and immunoblotting. A is control, B is ROT treatment.

Treatment of BmN4 cells with 50 µM ROT produced a shift in the pI to acidic, as shown on 2D-PAGE and immunoblotting (Fig. 5).

### BmDJ-1 overexpression in larvae causes resistance to ROT

**Figure 6:**
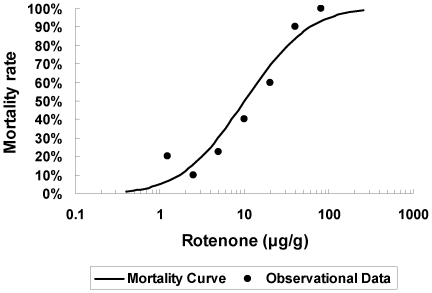
Dose mortality curve of rotenone in the silkworm. ROT (0, 1.25, 2.5, 5.0, 10, 20, 40, and 80 µg/g) was injected in day 3 fifth instar larvae, and the mortality rate within 24 h was examined. Filled circles were observational data.

ROT was used to produce an oxidative stress in order to examine the effect of exogenous BmDJ-1 protein. We determined the lethal dose (LD) of ROT for day 3 fifth instar larvae of 10.1 µg/g (LD_50_; 95% CI, 6.02–17.4) (Fig. 6). Based on computer simulations of reactivity using SAS software, we determined the optimal ROT concentration for further testing of the protective effects of BmDJ-1 to be 20 µg/g.

A BmDJ-1 overexpressing silkworm produced by injecting day 0 fifth instar larvae with recombinant BmNPV-BmDJ-1 virus showed significantly decreased mortality following intrahemocoelical injection of 20 µg/g ROT after 4 days (day 3 fifth instar larvae). For three experiments, the BmDJ-1 group mortality of 38, 27, and 15% was significantly lower than mortality rates of the blank vectors (control) of 81, 93, and 90%, respectively (P<0.01, <0.001, <0.001, respectively; Table 1).

**Table 1:** Mortality rate of BmDJ-1 overexpressing (rBmNPV-infected) silkworm exposed to ROT oxidative stress.

	Silkworm mortality^a^ (%)	
Experiment	Control	BmDJ-1
1	17/20 (85)	8/21 (38)**
2	14/15 (93)	4/15 (27)***
3	18/20 (90)	3/20 (15)***

a Rotenone (20 µg/g) was injected into BmDJ-1 overexpressing day 3 fifth instar larvae as shown in Fig. 7, and the mortality rate (dead silkworms/total silkworms; mortality rate, % in parentheses) within 24 h was examined.

**P<0.01,

***P<0.001 compared with control values.

We also confirmed virus-derived BmDJ-1 expression levels in the fatbodies of several insects after 1 day (24 h) and 4 days (day 4 fifth instar larvae) by RT-PCR and after 4 days by immunoblotting. Expression of blank-vector recombinant virus was detected as a 300-bp band, while virus-derived BmDJ-1 expression was detected as an 850-bp band. The BmDJ-1 expression was absent at 24 h but was detected at 96 h (Fig. 7A). Virus-derived BmDJ-1 protein was expressed at about 2-fold greater levels in non-infected groups after 4 days, and BmDJ-1 protein expression in blank virus-infected control groups was significantly decreased (Fig. 7B and S2).

**Figure 7:**
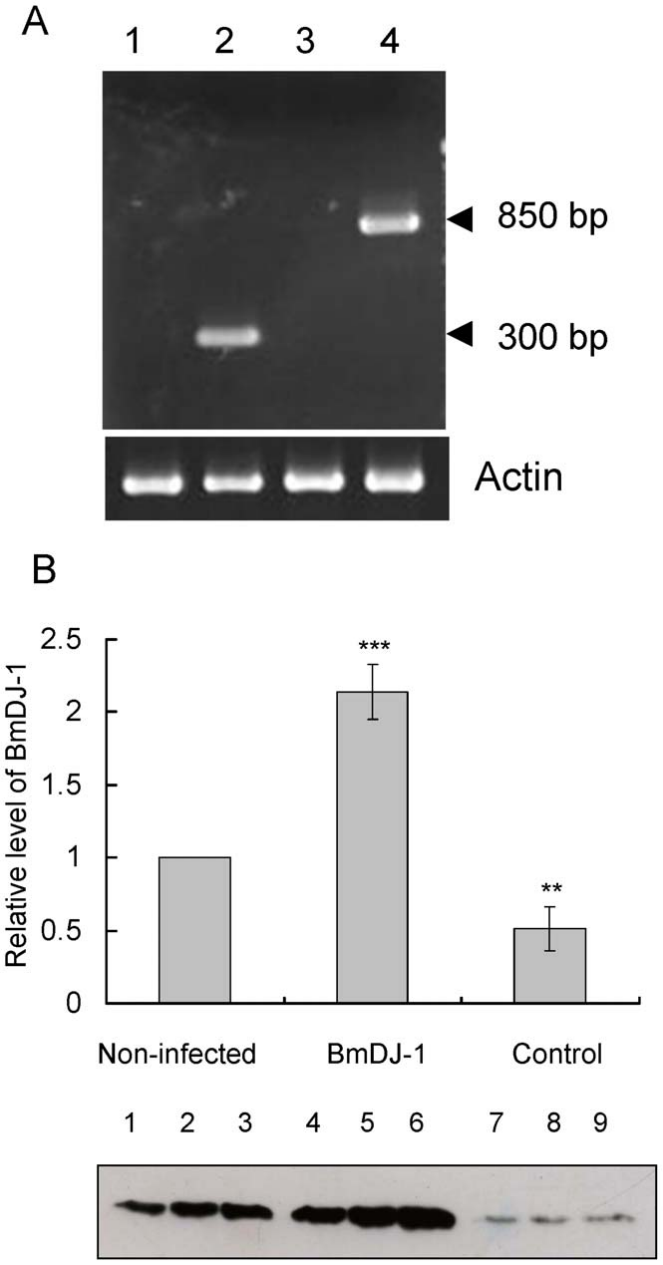
Expression of BmDJ-1 in silkworms infected by recombinant BmNPV. A. The fatbodies of several insects were dissected after 1 day (24 h) and 4 days (day 3 fifth instar larvae) and subjected to RT-PCR with BmNPV specific primers. Lanes 1, 2: blank virus; lanes 3, 4: recombinant virus; lanes 1, 3: 1 day after infection; lanes 2, 4: 4 days after infection. B. Aliquots (5 µg) of protein samples from fatbodies of several insects separated by SDS-PAGE, transferred to nitrocellulose, and probed with anti-BmDJ-1 antibody: non-infected control (day 3 fifth instar larvae) from experiments 1 (lane 1), 2 (lane 2) and 3 (lane 3); infected by recombinant virus from experiments 1 (lane 4), 2 (lane 5), 3 (lane 6); and blank virus after 4 days infection from experiments 1 (lane 7), 2 (lane 8), and 3 (lane 9). Protein level was measured by Image J ver 1.37 c and plotted to a graph. *P<0.05, **P<0.01 compared with control values.

### Expression of BmDJ-1 and NO concentration

**Figure 8:**
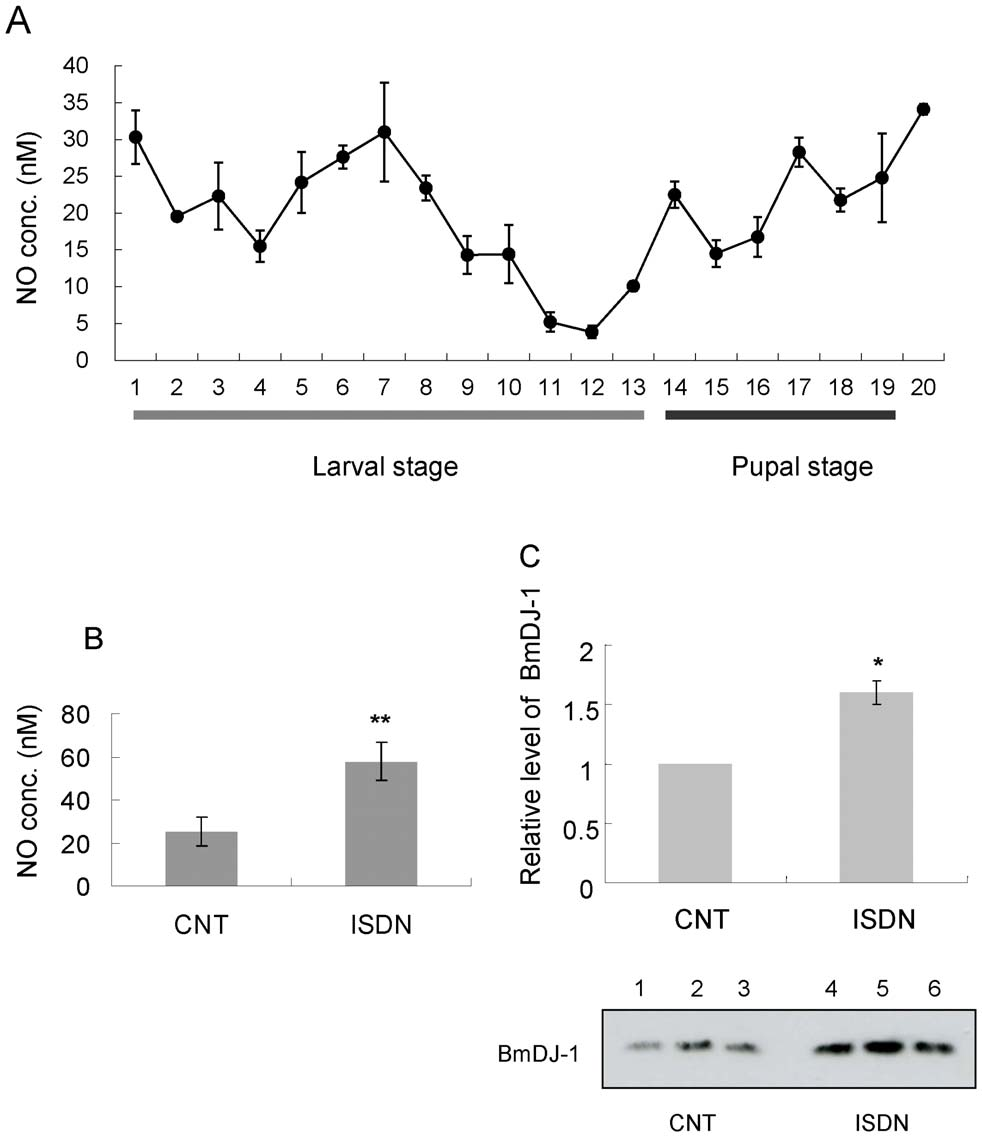
Change of NO concentration in hemolymph and expression of BmDJ-1 treated with ISDN. A. The hemolymph of several insects was collected from day 0 fifth instar larvae to adults and measured for NO concentration. 1–13: fifth instar larval stage (day 0 to 12); 14–19: pupal stage (day 0, 1, 3, 4, 7, 8); 20: adult. B. NO concentration in the medium. C. Aliquots (5 µg) of protein samples from BmN4 cells, experiment 1 of control (lane 1), experiment 2 of control (lane 2), experiment 3 of control (lane 3), experiment 1 of ISDN treatment (lane 4), experiment 2 of ISDN treatment (lane 5), and experiment 3 of ISDN treatment (lane 6) were separated by SDS-PAGE, transferred to nitrocellulose, and probed with anti-BmDJ-1 antibody. Protein level was measured by Image J ver 1.37 c and plotted to a graph. *P<0.05, **P<0.01 compared with control values.

The expression pattern of BmDJ-1 was tissue-specific, reflecting the unique responses to oxidative stress. We examined some factors that might affect expression. NO concentration in the hemolymph was found to fluctuate from the fifth instar larva to adult (Fig. 8A), with high levels for day 0 and 6 fifth instar larvae and adults and gradually increasing NO concentration in the pupal stages. To test whether NO affects the expression of BmDJ-1, BmN4 cells were treated with 100 µM ISDN as an NO donor for 16 h. BmDJ-1 was detected in each sample by SDS-PAGE and immunoblotting with NO concentration (Fig. 8B) and BmDJ-1 expression (Fig. 8C and S3) increased compared to the control (0.1% ethanol).

## Discussion

Throughout its evolutionary history, DJ-1 shows a highly conserved amino acid sequence. Characterization of the *B. mori* variant, BmDJ-1, by cDNA cloning from the brains of the fifth instar larvae shows the presence of Cys and Leu, which are key residues for the function of DJ-1.

On a phylogenetic tree of DJ-1 proteins, two orthologs of *D. melanogaster*, DJ-1α and DJ-1β, and BmDJ-1 placed in distinct clusters. *D. melanogaster* DJ-1α is most highly expressed in the testis from the pupal stages to adult, and DJ-1β is expressed in almost all tissues from embryo to adult. Loss-of-function DJ-1β mutant flies are sensitive to oxidative modification from H_2_O_2_ and paraquat, although the role played by DJ-1α remains unclear [22]. Thus, these two *D. melanogaster* DJ-1s appear to have distinct functions.

In contrast, BmDJ-1 exists as a single isoform based on the single 756-bp transcript and only one band for BmDJ-1 on northern blot assay. The EST database (SilkBase; http://morus.ab.a.u-tokyo.ac.jp/cgi-bin/index.cgi) shows two distinct EST clones (data not shown). While BmDJ-1 may exist as several kinds of splice variants, this could not be clarified in this study.

### BmDJ-1 demonstrated resistance to oxidative stress by ROT

DJ-1 has been reported to play a role in anti-oxidative stress by several independent groups. We confirmed that BmDJ-1 changes to an acidic form that is affected by ROT treatment in BmN4 cells (Fig. 5), indicating a response to oxidative stress.

In exogenous tests of BmDJ-1 with ROT, the mortality rate of individuals with BmDJ-1 is significantly decreased in the presence of ROT treatment, while the control groups remain extremely sensitive. It has been reported that the start of protein synthesis for BmNPV is 24 h after infection and that the protein expression level peaks at 96 h [23]. Endogenous protein synthesis stopped at 24 h. Immunoblotting at 96 h showed that virus-derived BmDJ-1 protein expression was significantly increased and virus-infection control group of BmDJ-1 protein expression was significantly decreased. Our findings of virus-derived BmDJ-1 expression after 96 h corroborate those results and suggest that BmDJ-1 overexpression improves the survival of silkworm larvae treated with ROT.

### BmDJ-1 expression controlled with NO

BmDJ-1 showed a tissue-specific expression pattern that indicates unique responses to oxidative stress. We found that NO was an oxidative stressor in *B. mori* that could be modulated by BmDJ-1.

The BmDJ-1 expression pattern in tissues in this study suggested that BmDJ-1 expression correlates to the hemolymph NO concentration, which showed day-to-day fluctuation from fifth instar larvae to adult (Figs. 4C and 8A). Moreover, the expression of BmDJ-1 was increased and the pI shifted acidic due to exposure to an NO donor (data not shown). These results showed that BmDJ-1 was oxidized and its expression was regulated by NO.

Choi et al. [24] reported that the nitric oxide synthase (NOS) gene in *B. mori* shows the highest expression in Malpighian tubule in day 7 fifth instar larvae, suggesting that NO might be related to *B. mori* metamorphosis. Inoue et al. [25] reported that administration of ISDN, an NO donor, to the beetle *Homoderus mellyi parry* rapidly progresses pupation. Conversely, the administration of carnitine, which suppresses apoptosis of cells in larval beetles, extended the larval developmental period and generated huge adult beetles. These observations implicate NO in the mechanism of metamorphosis as an apoptosis initiator, though the underlying process remains unclear.

In *B. mori*, apoptosis is the principal mechanism for dynamic remodeling of the body structure during metamorphosis. Apoptosis mainly occurs during the pupal developmental period, during which the restructuring produces the adult body. In our observation, the increased expression levels of BmDJ-1 occur in the pupal developmental stage, which coincides with apoptosis and the apparent melting of the body. BmDJ-1 might be involved in the elimination of NO in metamorphosis.

Although DJ-1 protein acts as a controller caspase activation to alter self expression level in the apoptotic pathway [26], we cannot determine a direct relationship between BmDJ-1 and NO generation in metamorphosis based on these experiments.

In future studies, we will investigate whether BmDJ-1 directly regulates NO in metamorphosis.

## Materials and Methods

### Ethics statement

The study protocol for the experimental use of the animals was approved by the Ethics Committee of Meiji Pharmaceutical University (Approval ID 2004).

### Insects

The hybrid strain Kinshu x Showa was supplied from Ueda-Sha Co. Ltd., Nagano, Japan. Individuals were reared on the artificial diet Silkmate 2S (NOSAN, Tsukuba, Japan) and kept at 25°C on a 12 h light/12 h dark daily cycle.

### Cell culture

An established silkworm cell line, BmN4 (NOSAN), was maintained at 25°C in TC-100 medium (NOSAN) supplemented with 10% fetal bovine serum and Antibiotic-Antimycotic (Invitrogen, Carlsbad, CA, USA).

### Molecular cloning of BmDJ-1

We first searched the *B. mori* expressed sequence tag (EST) database on KAIKOBLAST (kaikoblast.dna.affrc.go.jp) using the *Drosophila melanogaster* DJ-1 alpha (NM_137072) or beta (NM_143568) sequence as a query, and identified the EST clone NRPG1136, which did not overlap the 5′ end of the coding region of the *B. mori* DJ-1 gene (BmDJ-1). The entire coding sequence was determined using total RNA extracted from the brains of day 3 fifth instar larvae by an RNeasy mini kit (Qiagen, Valencia, CA, USA). DNase-treated total RNA was processed for cDNA synthesis using oligo(dT)12-18 primers and SuperScript II reverse transcriptase (Invitrogen), and cDNA was amplified by PCR using Pfu Turbo DNA polymerase (Stratagene, La Jolla, CA, USA) and the primers 5′-TCAAGAACAATGAGCAAGTCTGCG-3′ and 5′-TAATATTAGTACTGCGAGATTAAC-3′. The amplified products were cloned into a cloning vector p3T (MoBiTec, Göttingen, Germany). The purified vectors were processed for sequencing by the dideoxynucleotide chain termination method on an ABI PRIZM 3100 Genetic Analyzer (Applied Biosystems, Tokyo, Japan). The cDNA clone, NRPG1136, was provided by the National Bioresource Project (MEXT, Japan).

### 5′-Rapid Amplification of cDNA ends

The 5′-terminal cDNA ends were amplified using the SMART RACE cDNA Amplification kit (Clontech, Mountain View, CA, USA) according to the supplier's instructions with primers 5′-GCCAGCTAGAGTAACTGTTACCCC-3′ and 5′-AGTCACTTGCCTTGAGCACAGCAC-3′. The amplified products were cloned into a p3T vector for sequencing.

Deduced amino acid sequences, were aligned and phylogenetic trees and homology analyses were done using BLAST (blast.genome.jp), CLC Free Workbench ver 3.2.3 (CLC Bio, Aarhus, Denmark), and Genetyx ver 9.0 (Genetyx Co., Tokyo, Japan).

### Recombinant protein

The ORFs of BmDJ-1 were amplified by PCR using PfuTurbo DNA polymerase and primers 5′-AGCAAGTCTGCGTTAGTGAT-3′ and 5′-TTAGTACTGCGAGATTAACA-3′. Products were cloned into a prokaryotic expression vector pTrcHis-TOPO with a TOPO TA cloning kit (Invitrogen) and expressed in *E. coli* as fusion proteins with N-terminal Xpress tags. The nucleotide sequence was confirmed by sequencing. Recombinant BmDJ-1 expressed in *E. coli* was purified with HIS-Select spin columns (Sigma, St. Louis, MO, USA) according to methods described previously [27]. A recombinant β-galactosidase (LacZ) fragment tagged with X-press included in the TOPO TA cloning kit was used as a negative control.

### Immunology

The antibody for immunoblotting was raised in Japanese white rabbits by subcutaneous injection of the recombinant BmDJ-1 and Ribi adjuvant system (Corixa Co., Hamilton, MT, USA) mixture. The serum was stored at −80°C.

### Immunoblotting

To identify the presence of BmDJ-1 in different tissues and cells, protein samples (5 µg) were separated on SDS-PAGE, transferred to nitrocellulose membranes using the method of Towbin *et al*. [28], and immunoblotted using rabbit anti-BmDJ-1 antibody and goat anti-rabbit IgG-conjugated horseradish peroxidase (HRP). The membranes were developed using a chemiluminescent substrate (Pierce, Rockford, IL, USA).

The tissue distribution of BmDJ-1 was determined for the midgut, fatbody, Malpighian tubule, testis, and ovary from day 0 fifth instar larvae, pupae, and adults. Each tissue sample was run on the same gel, which was also loaded with 20 ng of recombinant Xpress-tagged BmDJ-1. The distribution of BmDJ-1 from first to fifth instar larvae, pupae and adult on the whole body and brain of larvae, pupae and adults were also determined. All tissues were homogenized in RIPA lysis buffer composed of 50 mM Tris-HCl, pH 7.5, 150 mM NaCl, 1% Nonidet P40, 0.5% sodium deoxycholate, 0.1% SDS, and a cocktail of protease inhibitors (Sigma), followed by centrifugation at 10,000× g for 15 min.

The protein concentration was determined by a Bradford assay kit (Pierce). Samples of supernatant (5 µg of protein) were separated by SDS-PAGE, transferred to nitrocellulose membranes, and immunoblotted with anti-BmDJ-1 antibody following the procedure described above.

### Specificity of antibody against BmDJ-1

We examined the specificity of antibody against BmDJ-1 using following samples: 0.25 or 0.5 µg recombinant BmDJ-1 protein with xpress tag, 1 µg recombinant CBP protein with GST tag [21], 10 µg HEK293 cell lysate, BmN4 cell lysate and 10 µg larva brain lysate.

### Northern blot analysis

Total RNA derived from the ovaries of day 4 fifth instar larvae were used. Total RNA (12 µg) was separated on a 1.5% agarose-6% formaldehyde gel and transferred to a nylon membrane. DIG-labeled probes were synthesized using the PCR DIG probe synthesis kit (Roche Diagnostics, Mannheim, Germany) according to the supplier's instructions with the primers 5′-CATTTGTGCTGCTTCCATAGCGTT-3′ and 5′-CATTCCCTTTTCGACTTGATCGGC-3′. After pre-hybridization, the membranes were hybridized with the DIG-labeled probes at 54°C overnight. The specific reaction was visualized on Kodak X-OMAT AR X-ray films by the DIG chemiluminescence detection kit (Roche Diagnostics).

### RT-PCR

Total RNA derived from the brain, midgut, fatbody, Malpighian tubule, testis, ovary, and hemocyte of day 4 fifth instar larvae was DNase-treated and processed for cDNA synthesis using oligo(dT)12–18 primers and SuperScript II reverse transcriptase (Invitrogen). cDNA was amplified by PCR using Taq DNA polymerase (Qiagen) and the primers 5′-CATTTGTGCTGCTTCCATAGCGTT-3′ and 5′-CATTCCCTTTTCGACTTGATCGGC-3′. Amplification was carried out for 30 cycles of denaturing for 40 s at 94°C, annealing for 40 s at 50°C and extension for 90 s at 72°C. Amplified PCR products were separated by agarose gel, stained with ethidium bromide, and visualized under UV light.

### Transfer plasmid and generation of recombinant virus

The ORF sequence of BmDJ-1 was amplified by PCR from brain cDNA as described above, with the primers 5′-GGGGTACCCCATGAGCAAGTCTGCGTTAGTGAT-3′ and 5′-GGAATTCCAATATTAGTACTGCGAGATTAAC-3′. The amplified region was digested with EcoRI and KpnI and cloned into the baculovirus transfer pBK283 vector. Blank pBK283 vector was used as a control. For generating recombinant BmNPV, we used a Bom-EX kit (NOSAN) according to the supplier's instructions. The recombinant BmNPV nucleotide sequence was confirmed by sequencing using the primers 5′-ACTGTCGACAAGCTCTGTCC-3′ and 5′-ACAACGCACAGAATCTAACGC-3′. Purified recombinant virus was titrated by plaque assay, and high titer stocks (2×10^7^ pfu/ml) were used for infecting larvae.

### Determination of LD_50_ of day 4 fifth instar larvae by ROT stimulation

To determine the LD_50_ of day 3 fifth instar larvae by ROT (Sigma) stimulation, we injected ROT intrahemocoelically to larvae weighing 3.5 to 4.0 g using a disposable syringe (Terumo, Tokyo, Japan) with a 30G needle. ROT was dissolved in DMSO (prepared immediately before use and stored in the dark) at 0, 1.25, 2.5, 5.0, 10, 20, 40, and 80 µg/g and injected into larvae in a volume of 10 µl/g body weight. The number of dead silkworms after 24 h was counted and the mortality rate (%)  =  (X/Y)×100 was calculated, where X =  dead larvae in the group and Y =  total larvae in the group. The mortality rates were analyzed with Probit analyses [29] using the Probit Analysis option in the SAS 8.2 software package (SAS Institute Japan Ltd., Tokyo, Japan) to calculate the LD_50_.

### Overexpression of BmDJ-1 to larvae and exposure to ROT oxidative stimuli

A 50 µl aliquot of BmNPV-BmDJ-1 or BmNPV-blank-vector (1×10^5^ pfu/larva) was injected intrahemocoelically into day 0 fifth instar larvae using a disposable syringe (Terumo) with a 30G needle. Blank-vector recombinant virus was injected as a control. After rearing for 4 days on an artificial diet, larvae were examined for overexpression of BmDJ-1 to assess protection from oxidative stress due to ROT.

Virus-derived BmDJ-1 expression level was measured in the dissected fatbodies of several insects after 1 day (24 h) and 4 days (day 3 fifth instar) by RT-PCR with the primers 5′-ACTGTCGACAAGCTCTGTCC-3′ and 5′-ACAACGCACAGAATCTAACGC-3′.

Virus-derived BmDJ-1 expression level was measured in the dissected fatbodies of several insects after 4 days (day 3 fifth instar) by immunoblotting.

We surmised the ROT dose that would be most effective in the experimental model with exogenous BmDJ-1 based on a report of the administration of exogenous DJ-1 [30].

ROT, prepared at 20 µg/g (LD_70_), was injected to three groups of 10 to 20 larvae in a volume of 10 µl/g body weight. The number of dead silkworms after 24 h was counted and the mortality rate (%) was calculated.

Data were analyzed with the multiple comparison test followed by the Cochran-Armitage test for dose-response relationship and Steel's (non-parametric) multiple comparison test. P<0.05 was considered significant. All statistical analyses were carried out using SAS system 8.2 software. Three trials were performed in each experiment.

### BmN4 cells treated with ROT, two-dimensional (2D) gel electrophoresis, and detection of BmDJ-1

BmN4 cells (2×10^6^) were grown on 6-well Falcon plates (BD Biosciences, Franklin Lakes, NJ, USA) and washed twice with PBS followed by 3 h of treatment with TC-100 medium containing ROT (50 µM) dissolved in 0.1% DMSO or 0.1% DMSO as a control in the dark. To prepare total protein extracts for two-dimensional (2D) gel electrophoretic analysis, the cells were sonicated in rehydration buffer comprising 8 M urea, 2% CHAPS, 0.5% carrier ampholytes at pH 3–10, 20 mM dithiothreitol, 0.002% bromophenol blue, and a cocktail of protease inhibitors. Urea-soluble proteins were separated by isoelectric focusing (IEF) using the ZOOM IPGRunner system loaded with an immobilized pH 3–10 gradient strip (Invitrogen), as described previously [16]. After the first dimension of IEF, the protein was separated in the second dimension on a 4–12% NuPAGE polyacrylamide gel (Invitrogen). For detection of BmDJ-1, the gel was transferred to a polyvinylidene difluoride (PVDF) membrane for immunoblotting.

All incubation steps were carried out at 25°C in the dark. Three trials were performed for each experiment.

### Collection of samples and measurement of NO levels

Hemolymph (250 µl) was collected from day 0 fifth instar larvae, pupae and adults, or from medium to measure the concentration of NO. To remove proteins, samples were mixed with methanol (2∶1 by volume), followed by centrifugation at 10,000× *g* for 20 min, and NO levels in the supernatants were measured using an NOx analyzer (ENO-20; Eicom, Kyoto, Japan), according to the manual.

### BmN4 cell treatment with ISDN and detection of BmDJ-1

BmN4 cells (2×10^6^) were grown on 6-well Falcon plates (BD Biosciences) and washed twice with PBS followed by 16 h of treatment with TC-100 medium containing 100 µM of isosorbide dinitrate (ISDN; prepared immediately prior to use and kept in the dark) dissolved in 0.1% ethanol or with 0.1% ethanol alone as a control. Total protein extracts were prepared for immunoblotting and culture medium was prepared for NO analysis. Statistical analysis was performed using Student's *t*-test. P<0.05 was considered significant. All statistical analyses were carried out using SAS system 8.2 software. Three trials were performed for each experiment.

## Supporting Information

Figure S1 **SDS-PAGE and CBB staining of** **figure 4** **.** A. AWhole body homogenates from day 0 larvae of the first (lane 1), second (lane 2), third (lane 3), fourth (lane 4), and fifth (lane 5) instars, the pupae (lane 6), and the adult (lane 7). B, Brain of the fifth instar larvae (lane 8), pupae (lane 9), and adults (lane 10). C. a, midgut; b, fatbody; c, Malpighian tubule; d, testis; and e, ovary were isolated from day 0 to 12 fifth instar larvae (lanes 1 to 13), from day 0, 1, 3, 4, 7 and 8 pupae (lanes 14 to 19), and from day 0 adults (lane 20). No samples were loaded in panel a, lanes 17, 18, 19, 20; panel c, lane 16; and panel e, lane 13.
(TIFF)

Figure S2 **SDS-PAGE and CBB staining of** **figure 7B** **.** Non-infected control (day 3 fifth instar larvae)from experiments 1 (lane 1), 2 (lane 2) and 3 (lane 3); infected by recombinant virus from experiments 1 (lane 4), 2 (lane 5), 3 (lane 6); and blank virus after 4 days infection from experiments 1 (lane 7), 2 (lane 8), 3 (lane 9).
(TIFF)

Figure S3 **SDS-PAGE and CBB staining of** **figure 8C** **.** Experiment 1 of control (lane 1), experiment 2 of control (lane 2), experiment 3 of control (lane 3), experiment 1 of ISDN treatment (lane 4), experiment 2 of ISDN treatment (lane 5), and experiment 3 of ISDN treatment (lane 6).
(TIFF)
