# Supplementary figures and images for: BmDJ-1 Is a Key Regulator of Oxidative Modification in the Development of the Silkworm, Bombyx mori
Source: PLoS One. 2011 Mar 24;6(3):e17683. doi: 10.1371/journal.pone.0017683 (PMC3063780; doi:10.1371/journal.pone.0017683)

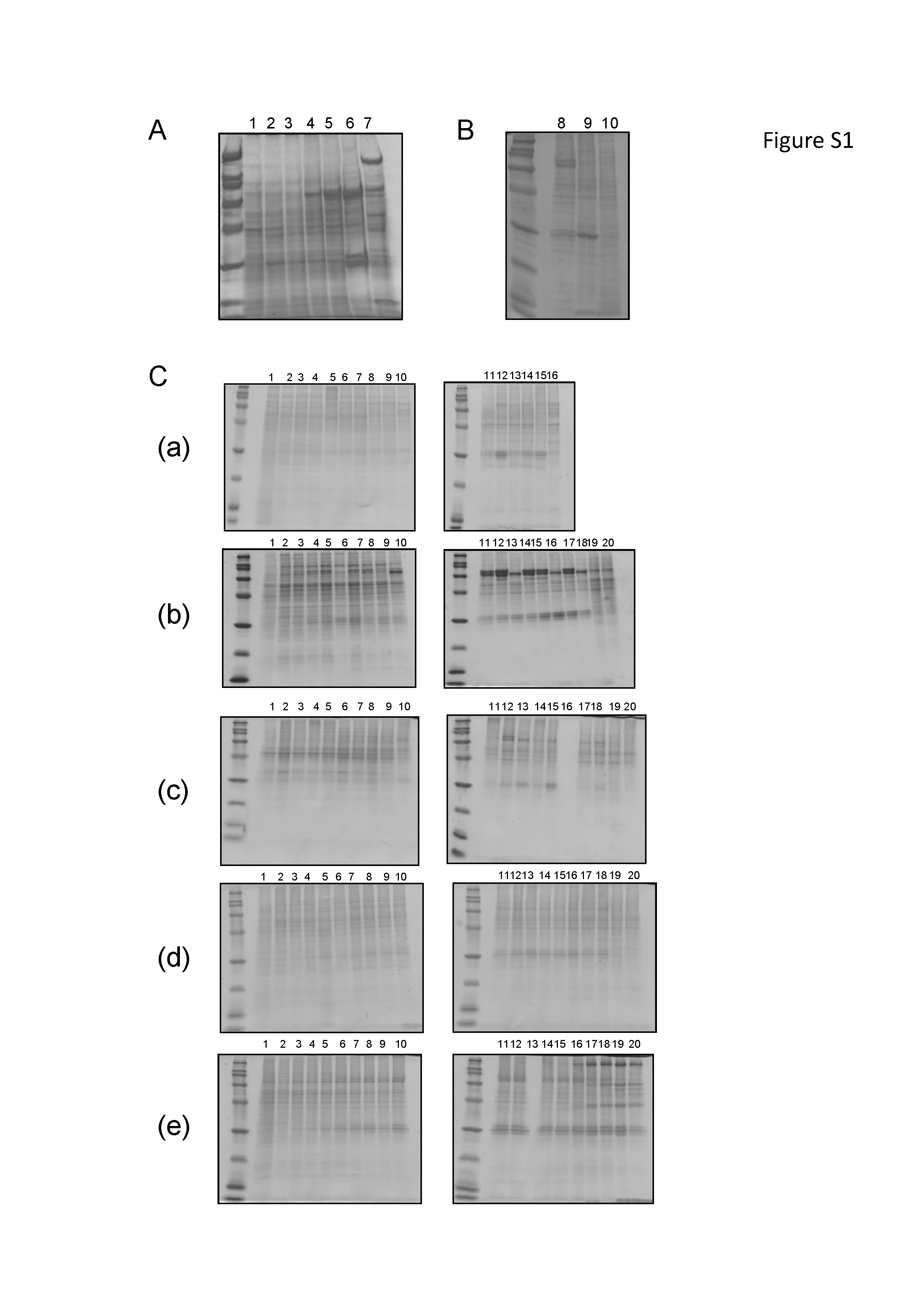

Supplement: Figure S1 — SDS-PAGE and CBB staining of figure 4 . A. AWhole body homogenates from day 0 larvae of the first (lane 1), second (lane 2), third (lane 3), fourth (lane 4), and fifth (lane 5) instars, the pupae (lane 6), and the adult (lane 7). B, Brain of the fifth instar larvae (lane 8), pupae (lane 9), and adults (lane 10). C. a, midgut; b, fatbody; c, Malpighian tubule; d, testis; and e, ovary were isolated from day 0 to 12 fifth instar larvae (lanes 1 to 13), from day 0, 1, 3, 4, 7 and 8 pupae (lanes 14 to 19), and from day 0 adults (lane 20). No samples were loaded in panel a, lanes 17, 18, 19, 20; panel c, lane 16; and panel e, lane 13. (TIFF) [file pone.0017683.s001.tiff]

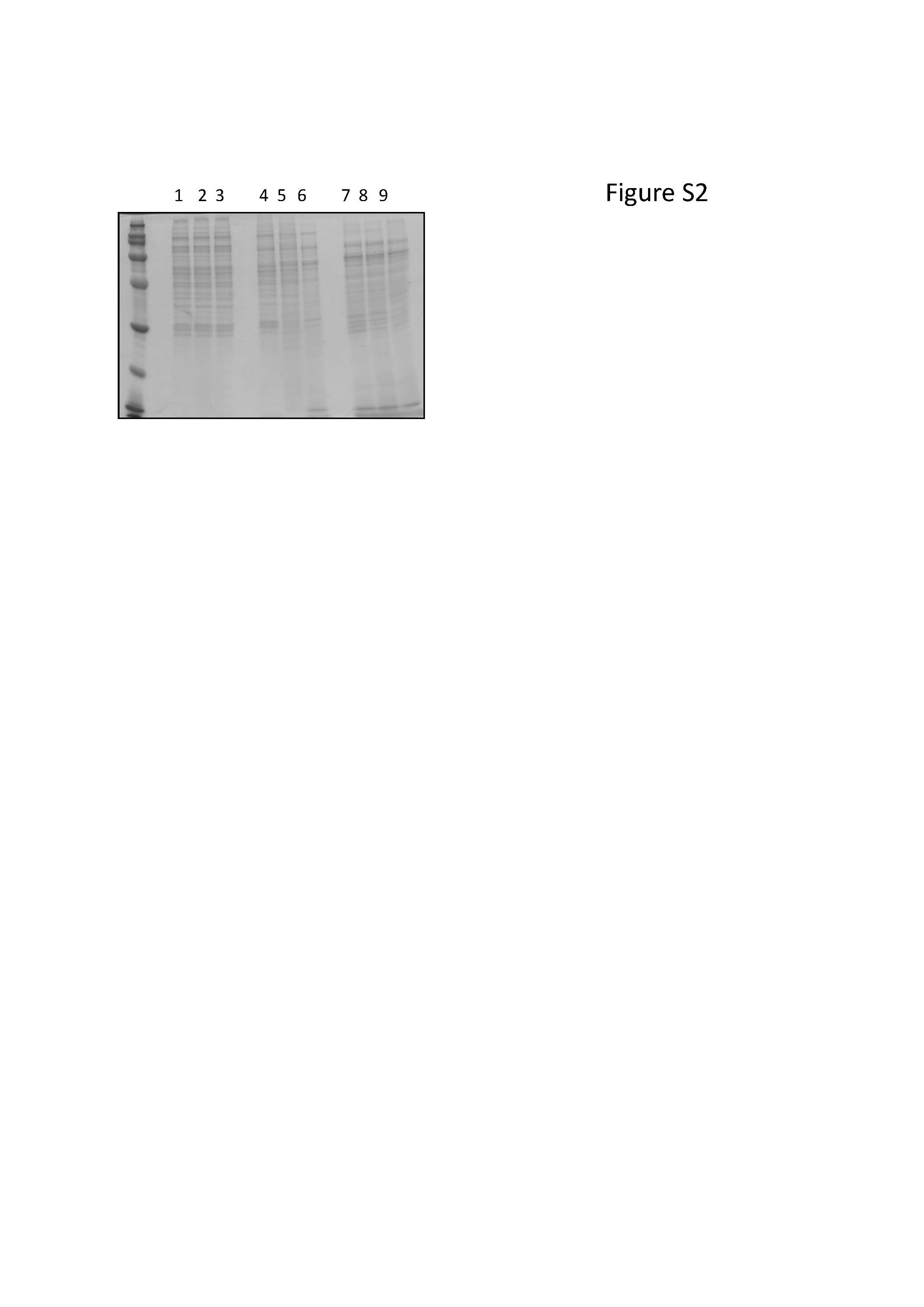

Supplement: Figure S2 — SDS-PAGE and CBB staining of figure 7B . Non-infected control (day 3 fifth instar larvae)from experiments 1 (lane 1), 2 (lane 2) and 3 (lane 3); infected by recombinant virus from experiments 1 (lane 4), 2 (lane 5), 3 (lane 6); and blank virus after 4 days infection from experiments 1 (lane 7), 2 (lane 8), 3 (lane 9). (TIFF) [file pone.0017683.s002.tiff]

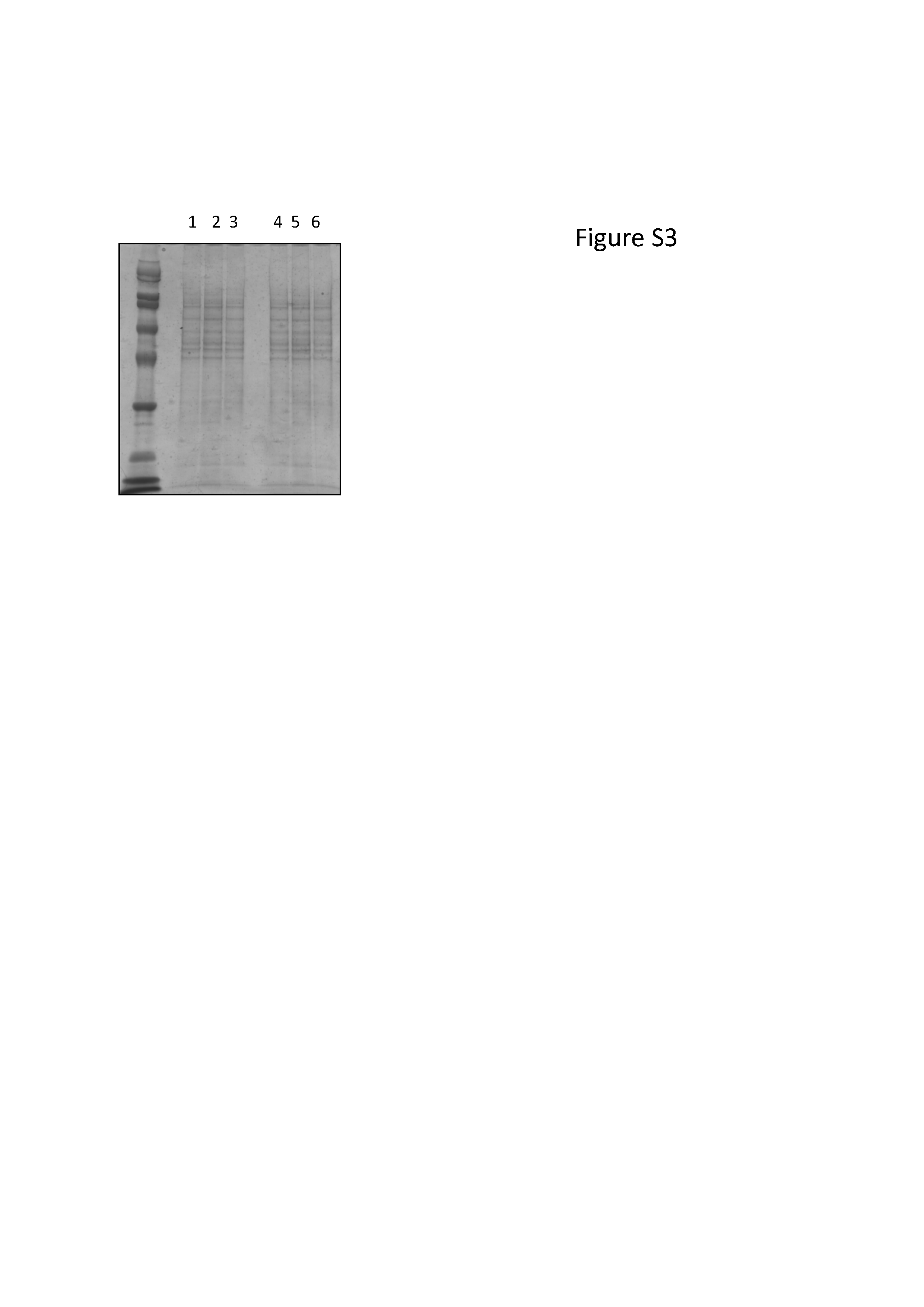

Supplement: Figure S3 — SDS-PAGE and CBB staining of figure 8C . Experiment 1 of control (lane 1), experiment 2 of control (lane 2), experiment 3 of control (lane 3), experiment 1 of ISDN treatment (lane 4), experiment 2 of ISDN treatment (lane 5), and experiment 3 of ISDN treatment (lane 6). (TIFF) [file pone.0017683.s003.tiff]
